# Crowdsourcing the identification of studies for COVID‐19‐related Cochrane Rapid Reviews

**DOI:** 10.1002/jrsm.1559

**Published:** 2022-04-25

**Authors:** Anna Noel‐Storr, Gerald Gartlehner, Gordon Dooley, Emma Persad, Barbara Nussbaumer‐Streit

**Affiliations:** ^1^ Cochrane Dementia and Cognitive Improvement Group, Radcliffe Department of Medicine University of Oxford Oxford UK; ^2^ Department for Evidence‐Based Medicine and Evaluation Danube University Krems Krems an der Donau Austria; ^3^ The RTI International‐University of North Carolina Evidence‐based Practice Center RTI International Research Triangle Park North Carolina USA; ^4^ Metaxis Ltd., Elmbank Offices Main Road Curbridge, Witney, Oxfordshire UK; ^5^ Department for Evidence‐Based Medicine Cochrane Austria, Danube University Krems Krems Austria

## Abstract

**Background:**

Utilisation of crowdsourcing within evidence synthesis has increased over the last decade. Crowdsourcing platform Cochrane Crowd has engaged a global community of 22,000 people from 170 countries. The COVID‐19 pandemic presented an opportunity to engage the community and keep up with the exponential output of COVID‐19 research.

**Aims:**

To test whether a crowd could accurately assess study eligibility for reviews under time constraints. Outcome measures: time taken to complete each task, time to produce required training modules, crowd sensitivity, specificity and crowd consensus.

**Methods:**

We created four crowd tasks, corresponding to four Cochrane COVID‐19 Rapid Reviews. The search results of each were uploaded and an interactive training module was developed for each task. Contributors who had participated in another COVID‐19 task were invited to participate. Each task was live for 48‐h. The final inclusion and exclusion decisions made by the core author team were used as the reference standard.

**Results:**

Across all four reviews 14,299 records were screened by 101 crowd contributors. The crowd completed each screening task within 48‐h for three reviews and in 52 h for one. Sensitivity ranged from 94% to 100%. Four studies, out of a total of 109, were incorrectly rejected by the crowd. However, their absence ultimately would not have altered the conclusions of the reviews. Crowd consensus ranged from 71% to 92% across the four reviews.

**Conclusion:**

Crowdsourcing can play a valuable role in study identification and offers willing contributors the opportunity to help identify COVID‐19 research for rapid evidence syntheses.

## 1 BACKGROUND

The COVID‐19 pandemic highlighted the need to produce reliable syntheses of health evidence as quickly as possible. An unprecedented volume of research has been undertaken resulting in a ‘tidal wave’ of trials and research publications.^1^ This infodemic makes the production of reliable health evidence synthesis especially challenging when it is needed most. Timely dissemination of accurate information is critical in the fight against both COVID‐19 and the harmful spread of mis‐information.^2^ Many questions have arisen regarding mechanism, transmission, diagnosis, prognosis, treatment and management of COVID‐19. In response to this global crisis, Cochrane launched a Rapid Review initiative (https://www.cochrane.org/cochranes‐work‐rapid‐reviews‐response‐covid‐19). Rapid Reviews are needed urgently to assess and appraise both existing actionable literature (on areas such as transmission mitigation, oxygen therapy, respiratory failure, and others) and to assess and appraise the exponentially growing corpus of research being produced as a direct result of COVID‐19.^3^

Crowdsourcing may help solve this data deluge challenge. Crowdsourcing is the outsourcing of needed tasks or activities to a large community of people, usually via the internet. Many domains and disciplines have implemented a range of crowdsourcing models to solve organisational or research problems. In psychology for example, crowdsourced research methods have been applied to overcome challenges of small sample sizes and enable research replication.^4^, ^5^ Crowds have also been engaged in helping to classify or categorise large amounts of data, from assessing underwater images from the Great Barrier Reef to helping to classify galactic data as part of the Galaxy Zoo citizen science project.^6^

Cochrane has used crowdsourcing as a means of effectively identifying health evidence since 2014. To date, over 200,000 trials have been identified for Cochrane's Central Register of Controlled Trials via Cochrane Crowd (https://crowd.cochrane.org), Cochrane's citizen science platform. Cochrane Crowd has attracted over 22,000 contributors from 170 countries. Accuracy evaluations have shown that the crowd, when performing a task with an appropriate agreement algorithm, can achieve 99% accuracy in terms of the crowd's ability to correctly identify studies of interest (for example, randomised trials) and the crowd's collective ability to reject the records that should be rejected.^7^

In April 2019, Cochrane launched a workflow called Screen4Me. This workflow enables Cochrane review author teams to send search results to Cochrane Crowd. Prior to this the crowd had focused on identifying studies for central repositories, such as Cochrane's Central Register of Controlled Trials. The Screen4Me workflow requires the crowd to work to a given deadline, assessing search results for a specific review, in return for named acknowledgement in the review when it is published.^8^, ^9^

Rapid Reviews on COVID‐19 present us with two specific new challenges with regards to the feasibility of recruiting and using a crowd effectively. The first is that it is likely that many Rapid Reviews undertaken will not be reliant on evidence from randomised controlled trials (RCTs) due either to the research or clinical question not being appropriate for RCTs or to the current lack of completed RCTs in this area. Therefore, the crowd will need to be able to identify and assess a range of different study types and designs. They will also be required to perform a more topic‐based assessment of the search results for Rapid Reviews. This has been shown to be feasible in two recent pilot studies performed with the Cochrane Crowd community. In the first pilot, the crowd were tasked with performing a topic‐based assessment for potentially relevant studies for an RCT‐based systematic review and, in the second, to perform a topic‐based assessment for a review that sought to include a range of different study types, including qualitative and mixed studies. In both pilot studies the crowd performed with a very high degree of accuracy: 100% and 96% sensitivity respectively.^9^, ^10^ Beyond Cochrane Crowd, other feasibility studies exploring the role of crowdsourcing in study identification have produced similar results.^11^, ^12^ Mortensen and colleagues tasked a crowd, via Amazon Mechanical Turk, with assessing the search results for four systematic reviews. The reviews included a range of study types and designs including randomised controlled trials and diagnostic studies. The crowd was able to achieve high sensitivity (ranging from 96% to 99%) and moderate specificity (6881%).^11^ Nama and colleagues' validation study used data from six systematic reviews across a wide range of healthcare areas and similarly demonstrated the feasibility of engaging a crowd to perform citation screening to a high degree of accuracy.^12^

Our second challenge relates to time‐to‐task‐completion. Rapid Reviews aim to be produced within a few weeks, with the results screening stage needing to be completed within 24 to 48 h. Cochrane's current Screen4Me workflow allows the crowd 2 weeks to complete the results screening task. This deadline is met for 95% of Screen4Me tasks.^13^ This is encouraging, but 2 weeks is a substantial increase on the hoped for 24 to 48 h for task completion for Rapid Reviews. The shorter timeframe therefore needs to be tested within the context of Rapid Reviews for COVID‐19, especially given that the task itself is different (as described above). In addition, time and accuracy are not mutually exclusive; one may adversely impact the other. Time pressure may increase crowd inaccuracy or reduce consensus (the proportion of records that do not require arbitration to reach a final decision) or both. We need to explore these factors in order to be able to better understand the role the crowd could play in the production of Rapid Reviews in this area.

## 2 AIMS AND OBJECTIVES

Our aim was to test whether a crowd could accurately assess the eligibility of search results for a range of Rapid Reviews when given a short deadline to do so. Our main outcome measures were time taken, in hours, to complete each of the screening tasks and time taken to prepare the customised training modules and other guidance materials required for each task. Additionally, we sought to measure crowd accuracy in terms of crowd sensitivity, specificity and crowd consensus.

## 3 METHODS

### 3.1 The datasets

We conducted a crowdsourced screening exercise using the sets of search results identified from a convenience sample of four Cochrane Rapid Reviews produced in response to the COVID‐19 pandemic. The four reviews were:

- Quarantine alone or in combination with other public health measures to control COVID‐19 (hereafter shortened to: *Review 1: Quarantine*)*^14^
- Barriers and facilitators to healthcare workers' adherence with infection prevention and control (IPC) guidelines for respiratory infectious diseases (*Review 2: IPC Adherence*)^15^
- Universal screening for Severe Acute Respiratory Syndrome Coronavirus 2 (*Review 3: Universal Screening*)^16^
- Convalescent plasma or hyperimmune immunoglobulin for people with COVID‐19 (*Review 4: Convalescent Plasma*)^17^

The size of the search results sets varied with the smallest being the set for Review 4: Convalescent Plasma (948 records) to the largest set for Review 1: Quarantine (5606). The inclusion criteria in terms of eligible study designs also varied across the four reviews. Review 1: Quarantine, included mathematical modelling studies, as well as interventional and observational study types. Review 2: IPC Adherence, included qualitative and mixed methods studies. Review 3: Universal Screening, included diagnostic test accuracy designs as well interventional studies as it considered both the accuracy and effectiveness of universal screening approaches, and Review 4: Convalescent Plasma, included both observational and interventional designs (see Table 1 for review characteristics). The final inclusion and exclusion decisions of studies made by the core author team for each of the four reviews was used as the reference standard. The screening process in place for Rapid Reviews differs slightly from the process for mainstream Cochrane systematic reviews in that records need only one assessment from a member of the core author team unless the record is rejected; rejected records are dual‐screened.^3^

**TABLE 1:** Key task characteristics

Review	Eligible study types	Size of set	No. of included studies^a^	No. of people invited	No. of people contributed	No. of records assessed/person (range)
Review 1: Quarantine	Observational modelling interventional	5606	47	123	65	4–1201
Review 2: IPC Adherence	Qualitative observational interventional	3367	32	85	36	2–1500
Review 3: Universal Screening	Observational (diagnostic) interventional	4378	18	104	38	10–3168
Review 4: Convalescent Plasma	Observational interventional	948	12	122	12	1–711
Total		14,299	109	287^b^	101^b^	268^c^

^a^ No. of included studies used in the evaluation datasets (some includes studies were used in the training modules so were not then included in the evaluation datasets).

^b^ Unique contributors.

^c^ Mean number of records assessed per crowd contributor.

### 3.2 The process

**FIGURE 1:**
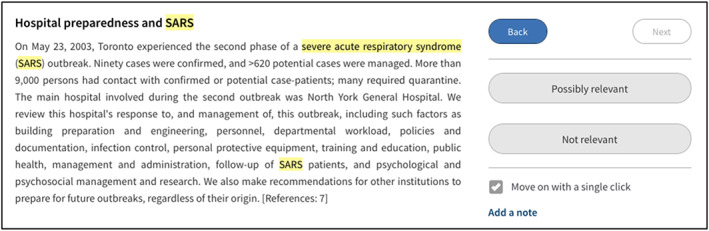
Screen shot of Review 1: Quarantine [Colour figure can be viewed at wileyonlinelibrary.com]

We created four separate tasks in Cochrane Crowd. With each, the crowd was tasked with classifying the search results based on an assessment of title‐abstract records (see Figure 1). We created a brief training module to accompany each of the four crowd tasks. Each module was composed of a series of introductory screens describing the topic of the review and the types of eligible studies followed by an assessment made up of sixteen practice records. We included two title‐only records within the training module for each review to help contributors know how to assess records that did not have abstracts. Crowd contributors needed to pass the assessment with a score of 80% or more to be able to progress to the live task. This pass mark is the standard pass mark used for other citation screening tasks in Cochrane Crowd. In addition to the training module, we employed an agreement algorithm which required three consecutive agreement classifications on a record for that record to be deemed either *Not relevant* (in the case for three independently made *Not relevant* classifications) or *Possibly relevant* (three consecutively made *Possibly relevant* classifications). We set each task to run initially for 48 h, with the option to extend the time if needed.

### 3.3 The crowd

Eligible crowd contributors were those who had completed and passed the training module for another task available in Cochrane Crowd: *COVID Quest*. *COVID Quest* was launched in May 2020.^18^ The task was built to help feed the Cochrane COVID‐19 Study Register (https://covid-19.cochrane.org). For this task, contributors need to be able to identify COVID‐19 related research as described by a title and abstract, and to then tag that research by study type and design, as well as assign study aims (e.g. treatment and management, or diagnostic, etc.). They must pass the *COVID Quest* training module by 80% or more to gain access to the live task.^19^ Once each rapid review crowd task had been built, contributors who had assessed at least one record in *COVID Quest* within the last month were contacted by email to inform them that they were eligible to participate in these Rapid Review tasks.

### 3.4 Data collection and statistical analysis

Crowd sensitivity was measured as the proportion of records correctly and collectively identified as *Possibly relevant* and crowd specificity, the proportion of records correctly and collectively identified as *Not relevant* to the review. We used the final set of studies included/not included in the review as the reference standard.

In terms of accuracy, we are primarily interested in crowd sensitivity rather than crowd specificity. The crowd missing or rejecting studies that should have been included is of more significance than the crowd mistakenly classifying irrelevant records as possibly relevant.

Crowd consensus is the proportion of records that the crowd assesses that do not require arbitration due to disagreeing classifications.

$$\frac{\mathrm{No}.\;\text{of records not requiring resolution}}{\text{Total number of records in dataset}}$$

We conducted all statistical analyses in Microsoft Excel v16.50 and SPSS v26.

## 4 RESULTS

### 4.1 Crowd characteristics

We created and ran four Cochrane Crowd tasks, one for each of the Cochrane Rapid Reviews used for this pilot study.^14^, ^15^, ^16^, ^17^ Table 1 shows, for each of the tasks, the number of contributors invited to take part, the number that took part, the size of each dataset and the time taken to complete the task. Eligible Crowd contributors were those who had taken part in the Cochrane Crowd task, *COVID Quest* within the last month prior to the date the rapid review task went live on the platform. For the Review 1: Quarantine, 65 crowd participants took part; Review 2: IPC Adherence, 36; Review 3: Universal Screening, 38; Review 4: Convalescent Plasma, 12. Of those who took part, 65% took part in only one of the tasks; the remainder (35%) took part in more than one. Crowd contributors screened on average 268 records; the ranges for each of the four reviews can be seen in Table 1.

### 4.2 Time

**FIGURE 2:**
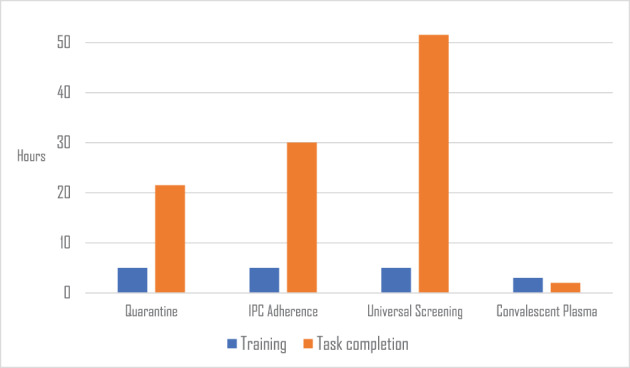
Outcome measure: Time [Colour figure can be viewed at wileyonlinelibrary.com]

Our main outcome measure was time, both in terms of time taken to produce the bespoke training modules and time to task completion by the crowd. Figure 2 shows the time taken to develop each training module, which ranged from 3 to 5 h, and the time‐to‐task‐completion, which ranged from 2 to 51.5 h. Time per 100 records for each of the reviews was therefore 22 minutes for Review 1: Quarantine, 53 minutes for Review 2: IPC Adherence, 74 minutes for Review 3: Universal Screening, and 13 minutes for Review 4: Convalescent Plasma.

### 4.3 Crowd accuracy: sensitivity and specificity

In terms of crowd accuracy, sensitivity (i.e., the crowd's collective ability to correctly identify the included studies) ranged from 94% to 100% (see Table 2). In Review 1: Quarantine, two included studies were missed by the crowd. In Review 2: IPC Adherence and Review 3: Universal Screening, one included study was incorrectly rejected. In Review 4: Convalescent Plasma, no included studies were missed.

**TABLE 2:** Crowd accuracy

Review	*N*	TP	TN	FP	FN	Sensitivity	Specificity	Consensus
Review 1: Quarantine	5606	45	3942	1617	2	95.7	70.9	72.02
Review 2: IPC Adherence	3367	31	2437	897	1	96.9	73.0	74.96
Review 3: Universal Screening	4378	17	3075	1285	1	94.4	70.5	71.34
Review 4: Convalescent Plasma	948	12	827	109	0	100.0	88.7	92.19

*Note*: TP = True Positive, the number of records correctly classified as possibly relevant; TN = True Negative, the number of records correctly classified as not relevant; FP = False Positive, the number of records incorrectly classified as possibly relevant; FN = False Negative, the number of records incorrectly classified as not relevant.

Crowd specificity (i.e., the crowd's collective ability to correctly reject ineligible references to studies) for each of the four reviews was: Review 1: Quarantine 71%, Review 2: IPC Adherence 73%, Review 3: Universal Screening 71%, and Review 4: Convalescent Plasma 89% (see Table 2).

### 4.4 Crowd consensus

**FIGURE 3:**
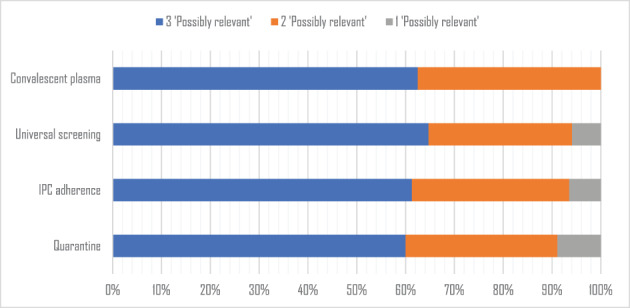
Crowd consensus for included studies [Colour figure can be viewed at wileyonlinelibrary.com]

The level of crowd consensus (i.e. the proportion of records receiving three consecutive agreeing classifications) was 72% for Review 1: Quarantine, 75% for Review 2: IPC Adherence, 71% for Review 3: Universal Screening, and 92% for Review 4: Convalescent Plasma (see Table 2). As well as evaluating crowd consensus for each data set as described above, we also calculated crowd consensus for just the eligible studies for each review. The proportion of included studies that received the required three *Possibly relevant* classifications was similar across all four reviews: Review 1: 60%, Review 2: 61%, Review 3: 65% and Review 4: 63% (See Figure 3).

### 4.5 Title‐only records

We explored whether records that did not have an abstract had an impact on accuracy or consensus measures. The proportion of title‐only records for each of the reviews was low (Review 1: 5.7%, Review 2: 7.2%, Review 3: 6.8%, Review 4: 6.6%). However, all four of the missed studies did have abstracts so this was not a factor in terms of negatively impacting crowd sensitivity. Where it did potentially have an impact on crowd performance is in terms of crowd consensus. Overall consensus ranged from 71% to 92% across each of the datasets. However, it was lower across both the eligible studies (range 60–65%) and lower still across records that did not have an abstract (54–61%). Neither finding is surprizing but both have implications for future potential applications of a crowd model for citation screening. The higher the prevalence of includable studies and/or the higher the proportion of title‐only records, the lower crowd consensus is likely to be.

## 5 DISCUSSION

The crowd performed three of the review tasks comfortably within the 48‐hour time limit, and one (Review 3: Universal Screening) in just over the time limit. This is an encouraging result. We had hoped to run the tasks either concurrently or in very quick succession to gauge the capacity of the crowd to handle multiple tasks simultaneously or continuously. However, we were unable to do that due to the availability of the datasets and the prioritisation of other COVID‐19 related activities. However, one advantage of having the tasks run approximately 4 weeks apart, meant that we were more likely to attract different crowd contributors for each task, giving us a better sense of generalizable crowd performance.

### 5.1 Analysis of missed studies

The crowd performed well across all reviews in terms of accuracy measures. Overall, out of a total of 109 included studies, the crowd incorrectly rejected four studies (3.7%). The titles of the four missed studies were:

1. Factors that make an infectious disease outbreak controllable^20^ (Review 1: Quarantine)
2. Severe Acute Respiratory Syndrome Coronavirus 2 Infection among Returnees to Japan from Wuhan, China^21^ (Review 1: Quarantine)
3. SARS: key factors in crisis management^22^ (Review 2: IPC Adherence)
4. Suppression of COVID‐19 outbreak in the Italian municipality of Vo, Italy^23^ (Review3: Universal Screening)

Two of the missed studies were from the quarantine review. One was a small modelling study pre‐dating the pandemic but deemed relevant in terms of modelling the effects of pre‐symptomatic infections. However, it provided only indirect evidence on SARS, not specifically on SARS‐CoV‐2. The other, an observational study, reported on the screening and quarantining of a cohort of Japanese nationals repatriated to Japan from Wuhan, China in early 2020. It may have been mistakenly perceived as a diagnostic study rather than of relevance to the quarantine measures review. The missed study from the IPC Adherence review was a qualitative study. It had very broadly stated aims to: “identify the key factors enabling the hospital to survive SARS unscathed.” The results described in the abstract make no direct mention of IPC Adherence but instead refer more broadly to good crisis management principles adopted by this specific hospital during the 2003 SARS epidemic. The final missed study was from the Universal Screening review (Review 3). It was not described explicitly as a screening study which may account for why it was missed.

Despite crowd sensitivity not achieving 100% for three of the four reviews used in this evaluation study, sensitivity was comparable to other similar studies run by this and other research teams^9^, ^10^, ^11^, ^12^ and potentially more accurate than having the search results screened by a single human assessor.^24^ However, it is arguable that providing a measure of sensitivity where the prevalence of included studies within each of the review datasets was very low, should be considered with caution: Review 1 had a prevalence of 0.87%, Review 2: 1.07%, Review 3: 0.53%, Review 4: 2%.

What is perhaps a more meaningful measure of performance is whether the conclusions of each review would have been altered by the missed studies. We contacted the lead authors for each of the reviews to ascertain whether conclusions would have changed. For Review 1: Quarantine, the missed studies would not have altered the conclusions of the review. The missed modelling study by Fraser and colleagues^20^ pre‐dated COVID‐19 and was based on SARS. This study therefore received less weight in the review's analysis than direct evidence based on SARS‐CoV‐2. The second missed study was deemed more important to the review. It was one of two observational studies on the quarantine of travellers. However, it would not have changed the direction of the finding nor the certainty of evidence grading (which was already very low). Therefore, missing this study would not have changed the review's conclusions. For Review 2: IPC Adherence, the missed study by Tseng and colleagues^22^ contributed to nine findings in the review. However, given the high number of other studies additionally contributing and the moderate to high confidence in these findings, it is likely the review would have drawn the same conclusions had the study not been included. Finally, for Review 3: Universal Screening, the missed study by Lavezzo and colleagues^23^ would also not have changed the conclusions nor the strength of the evidence for the findings it contributed to. The review author team noted within the review itself that the Lavezzo study did not contain specificity estimates and so had already analysed the effect of excluding this study, concluding that excluding it did not change the findings or range of estimates.^16^

As well as assessing the impact of missed studies, we also performed forward citation tracking to ascertain whether any of the missed studies would potentially have been retrieved via this method. This involves assessing the reference lists of included studies as a way of identifying additional studies missed by the electronic database searches. Of the four studies collectively rejected by the crowd, two were cited by other included studies in the reviews: one^20^ from Review 1: Quarantine, and the other^23^ from Review 3: Universal screening.

### 5.2 Impact of topic area

Another area of consideration is around whether domain or topic area affected crowd performance. One strength of this study was the range of review question types included: Review 1 was largely focused on observational and modelling studies (interventional designs were includable but unlikely to be found). Review 2 sought mixed methods studies and qualitative studies, Review 3, diagnostic and screening studies, and Review 4, interventional study designs. Research has highlighted the challenge in assessing studies for diagnostic‐related reviews,^25^, ^26^ and this appears to have been borne out in this evaluation study. In addition, no studies were incorrectly rejected for Review 4. This review sought to include studies that assessed the effectiveness of a treatment, convalescent plasma. This review was most alike other tasks hosted on Cochrane Crowd, namely the RCT identification task. This might account for the crowd's highly accurate and speedy performance.

### 5.3 Impact of agreement algorithm and training materials

Two other factors are also worth exploration in terms of possible impact on crowd accuracy: the agreement algorithm and the training materials. In terms of the agreement algorithm, we chose an algorithm (three consecutive agreements) that had produced high collective accuracy in other similar pilot projects.^9^, ^10^ Would altering the consecutive number of agreeing classifications have made a difference to collective accuracy? Starting with the accuracy of a single classification, the mean accuracy of individual contributors for each review was: 84.2% sensitivity and 82.2% specificity for Review 1; 86.6% sensitivity, 84.1% specificity for Review 2; 85.1% sensitivity, 89.9% specificity for Review 3; and 89.3% sensitivity, 90.9% specificity for Review 4. Taking the first two consecutive classifications made on each record across the four datasets would have resulted in reduced crowd sensitivity (in comparison to the ‘three agreement’ algorithm used for this study) with one additional study being missed per review. We do not have the data to model how an algorithm based on four consecutive agreeing classifications would have fared. However, interesting recent work by Nama and colleagues indicates that excellent sensitivity can be achieved with three assessments per record. In their analysis, increasing this number made little difference to sensitivity but decreased specificity.^27^

With regards to the training provided, we were able to provide highly representative records for the test set. We used a set of 16 records for each training module. In the recent evaluation by Nama and colleagues described above, the optimal size for the qualification set was explored. Their analysis indicated that the optimal size for a qualification set made up of true positives and true negatives was between 10–15 records.^27^

Despite this study's focus being on rapid reviews in the context of COVID‐19, the range of study types and designs eligible across the four reviews, and the correspondingly high levels of accurate screening by the crowd bode well for this approach being applied beyond a public health setting. Indeed, a recent overview by Burgard and colleagues describes initiatives underway to support ‘community‐augmented meta‐analyses’ in the field of psychology, leveraging distributed human effort to help curate the evidence base and produce ‘living’ or dynamic syntheses.^28^

This study has focussed exclusively on the use of crowdsourcing as a means of reliably expediting parts of the study identification stages of evidence synthesis. However, there is a growing field of research exploring the potential of machine learning for citation screening, for example using support vector machine learning classifiers that assign likelihood scores to records. The chief advantage of machine learning over crowdsourcing is time. Records can be classified by a machine learning classifier within minutes, irrespective of the size of the search results set; conversely a crowd will take a variable amount of time (though often still significantly faster than a small review author team). The significant challenge however with applying machine learning alone relates to the high‐quality training data required to build a reliable classifier. Also, for a machine learning classifier to operate as a binary classifier (replicating the human classification task), a calibration stage would be needed to ascertain the appropriate score threshold. Another approach, however, would be a hybrid machine‐crowd model. This might work well where there is limited training data or where sensitivity is paramount. One possible hybrid configuration would be to employ the classifier to help remove the more obviously not relevant material whilst engaging human effort to assess the remainder. This approach has been used to good effect in Cochrane in both its Screen4Me workflow and within Cochrane's broader Centralised Search Service initiative.^29^

Despite the safeguards described above, no system will be 100% accurate all the time. As well as quality control measures aimed at maximising crowd performance, review author teams also have a range of possible ways in which they can use the data generated by the crowd within their review production process. Table 3 presents three possible workflows regarding the use of the crowd's collective output, each dependent on the required outcome: sensitivity maximising (i.e. using the crowd in a way that reduces the risk of missing includable studies as much as possible), speed maximising, where time is the most critical factor and author team capacity is limited, or specificity maximising (reducing the number of false positives). The most appropriate approach will depend on the nature, complexity, and scope of the review itself, as well as the time and resources available to the author team.

**TABLE 3:** Crowdsourcing workflows

Sensitivity maximising	Crowd assessment + author team dual assessment of conflicting crowd records + author team single assessment of *Possibly relevant* records only
Speed maximising	Crowd assessment + author team single assessment of *Possibly relevant* records only
Specificity maximising	Crowd assessment + crowd resolver^a^ + author team single assessment of crowd identified *Possibly relevant* records only

a A crowd resolver is a crowd contributor assesses only records that have received discordant classifications, and makes a final crowd classification on the record.

## 6 CONCLUSION

This pilot study has demonstrated the feasibility of using a crowd in the study identification process for Cochrane Rapid Reviews. The crowd performed consistently well across each of the four evaluations in terms of time and accuracy measures. During a global health crisis, when time is of the essence and robust health evidence is critical, using crowdsourcing in this way offers a viable means to expedite the review process and offer willing contributors meaningful ways to get involved. The exact method of crowd application and use of crowd‐generated data will depend on the nature of the review itself and the urgency at which the evidence is required.

### Highlights

Over the last decade, crowdsourcing in health evidence synthesis has shown enormous potential, particularly in accurate and efficient study identification, as demonstrated by Cochrane Crowd.

This work adds to the growing evidence base regarding the capability of a crowd to identify studies accurately across a range of review questions when under time pressure.

This study focussed on Cochrane COVID‐19 related reviews but its findings indicate a much broader application for crowdsourcing in evidence synthesis, offering opportunities to speed up the review production process with minimal impact on quality.

## AUTHOR CONTRIBUTIONS

**Anna Noel‐Storr**: conceptualisation, methodology, investigation, data curation, visualisation, supervision, writing—original draft preparation. **Gerald Gartlehner**: conceptualisation, methodology, resources, data curation, writing—reviewing and editing. **Gordon Dooley**: conceptualisation, data curation, writing—reviewing and editing. **Emma Persad**: conceptualisation, data curation, writing—reviewing and editing. **Barbara Nussbaumer‐Streit**: conceptualisation, methodology, data curation, visualisation, writing—reviewing and editing.

## Data Availability

Data used in the study are available from: https://www.researchgate.net/publication/353372081_Rapid_review_evaluations_data_for_submission?_sg=CMmKYnkqK‐x3Kj7lP6r3YtXVvltK4cBQ7Y_AsIhLHL7iKHsFxL2‐UCYIM9d3g_bVWsuTL7cLM8Y0B6capYtf‐daykxivsVeT6Q791Fo7.o‐QlibkRRnt4yEvqnf5‐ArXSOTIU5VBWH8s84s95f9‐PH3RGoXgTHYMMTd7HZclqSvnQkDKuOKog5kMHAsTWNw
